# Physiological responses and intestinal conditions of broiler chickens treated with encapsulated *Acalypha australis* L. leaf extract and chitosan

**DOI:** 10.14202/vetworld.2024.994-1000

**Published:** 2024-05-09

**Authors:** Sugiharto Sugiharto, Yuki Zulpa, Ikania Agusetyaningsih, Endang Widiastuti, Hanny Indrat Wahyuni, Turrini Yudiarti, Tri Agus Sartono

**Affiliations:** Department of Animal Science, Faculty of Animal and Agricultural Sciences, Universitas Diponegoro, Indonesia

**Keywords:** broilers, chitosan, herbs, immune system, intestinal health, synergistic effect

## Abstract

**Background and Aim:**

The ban on antibiotic growth promoters adversely affects the physiological condition and health of poultry. The aim of this study was to determine the effect of encapsulated *Acalypha australis* L. leaf extract, chitosan, or a combination of both on the physiological and intestinal conditions of broiler chickens.

**Materials and Methods:**

A total of 280 Cobb broiler chicks were randomly distributed into four groups: Basal feed without additives (CNTL), basal feed with 0.01% encapsulated *A. australis* leaf extract (EALE), 0.01% chitosan (CHIT), and 0.01% EALE and 0.01% chitosan (EACH). Sample collection and data measurement were conducted on day 36.

**Results:**

There was a tendency (p = 0.08) for EACH bird to have a higher body weight than the other groups. Feed consumption was higher (p < 0.05) in EACH than in EALE and CHIT. Feed conversion ratio (FCR) was lower (p < 0.05) in EALE, CHIT, and EACH than in CNTL. Erythrocyte numbers were lower (p < 0.05) in EALE than in CNTL and EACH. Hematocrit was lower (p < 0.05) in EALE and CHIT groups than in the other two groups. There was a tendency (p = 0.09) for heterophils to be higher in EACH than in CNTL. Thrombocyte counts were lower (p < 0.05) in EACH group than in the other groups. Serum globulin levels were higher (p < 0.05) in EACH than in CNTL and CHIT. The albumin-to-globulin ratio was higher (p < 0.05) in CNTL than in EALE and EACH. Coliform bacteria tended to be lower (p = 0.05) in the cecum of EACH broilers than that of other broilers. Similarly, the ratio of Lactic acid bacteria to coliforms tended to be higher (p = 0.08) in the cecum of EACH group than that in the other groups. Treatments did not influence the intestinal morphology of broiler chickens (p > 0.05).

**Conclusion:**

A combination of EALE and chitosan as feed additives enhanced the final body weight and feed efficiency (FCR) of broilers. These additives also increased the levels of heterophils, serum globulin, the ratio of LAB to coliforms, and reduced thrombocytes, albumin-to-globulin ratio, and cecal coliform bacteria. Hence, EALE and chitosan blend improved the growth performance, immune status, and intestinal health of broiler chickens.

## Introduction

After the ban on antibiotic growth promoters (AGP), poultry nutritionists around the world searched for alternatives to AGP for broiler chickens. In relation to broiler chickens, many AGP substitutes have been investigated. However, the results have not consistently demonstrated that these AGP substitutes maximize the health and growth potential of broiler chickens. One of these AGP substitutes is herbal products. These broiler products have various active ingredients that function as immunomodulators, antibacterial agents, and antistress agents [1, 2]. *Acalypha australis* Linn leaves are one of these herbal products. The leaves of *A. australis* contain antibacterial active ingredients such as gallic acid [3]. *A. australis* leaves also contain alkaloids, saponins, and flavonoids, which have antioxidant and immunomodulatory properties [4].

In spite of its potential as an alternative to AGP, the use of herbal products is often hindered by the risk of damage to storage facilities. Under these circumstances, the use of herbal products as a substitute for AGP for broiler chicken may be ineffective. During storage, herbal products are often exposed to light, oxygen, and heat, which may damage the active components of the herbal product [5]. Low bioavailability of herbal products for the host is another factor that may limit their efficacy [6]. In addition, low absorption, low biotransformation, rapid metabolism, and rapid excretion can reduce the efficacy of herbal products as AGP substitutes [5, 6]. Nutritionists frequently extract and encapsulate herbal products to maximize their efficacy as feed additives [5, 7]. According to Sugiharto [7] and Natsir *et al*. [8], the use of encapsulated herbal extracts had a more prominent impact on the growth, intestinal microbial conditions, intestinal morphology, and physiological conditions of broiler chickens compared with the non-encapsulated extracts.

Chitosan, which is also often used as a feed additive for broiler chickens, is another alternative to AGP. Chitosan is a derivative of chitin and is mainly produced from the exoskeleton shells of arthropods (crabs and shrimp). Chitosan increases the growth rate of broiler chickens and improves their intestinal histomorphology, intestinal ecosystem, and physiological conditions [9, 10]. Tiyaboonchai [11] further revealed that chitosan can improve the host’s ability to absorb active compounds from the feed. In this study, *A. australis* leaves were extracted and encapsulated before being used as a feed additive for broiler chickens. The encapsulated *A. australis* leaf extract (EALE) was combined with chitosan to produce a synergistic effect.

The aim of this study was to determine the effect of EALE, chitosan, or a combination of both on the physiological and intestinal conditions of broiler chickens.

## Materials and Methods

### Ethical approval

The experiment was approved by the Animal Ethical Committee of the Faculty of Animal and Agricultural Sciences under approval number 59-07a/A-16/KEP-FPP.

### Study period and location

The study was conducted from April to July 2023 at the Faculty of Animal and Agricultural Sciences, Universitas Diponegoro, Semarang, Central Java, Indonesia.

### Preparation of encapsulated *A. australis* L. leaf extract

A. *australi*s leaves were collected from a garden close to the campus of Universitas Diponegoro and air-dried and milled into a fine powder. The leaves are identified and confirmed by the herbarium expert from the Department of Biology, Faculty of Sciences and Mathematics, Universitas Diponegoro. We soaked the leaf powder in 70% ethanol (1:6, g: mL) at room temperature (25°C) for 72 h. A vacuum rotary evaporator was used to evaporate the filtrate. Maltodextrin (1:1.25; g: g) was used to encapsulate the extract in accordance with the freeze-drying-based encapsulation technique. The EALE was kept refrigerated before use in *in vivo* experiments.

### Broiler chicken trials

A total of 280 mixed-sex Cobb broiler chicks were used in the experiment based on a fully randomized design. The chicks had an average body weight of 42.97 ± 1.64 g after weighing individually. Subsequently, they were divided into four groups of treatments. There were seven replications in each treatment group, with 10 chicks per replicate/pen. The treatment groups were as follows: (1) basal feed without feed additives (CNTL); (2) basal feed added with 0.01% EALE; (3) basal feed added with 0.01% chitosan (CHIT); and (4) basal feed with 0.01% EALE and 0.01% chitosan (EACH). The chicks were offered commercial pre-starter feed from the time of arrival until day 7, which, according to the feed label, contained 22–24% crude protein, less than 5% crude fiber, 5% crude fat, and 7% ash. Chicks were *ad libitum* fed starter and finisher feeds from days 8 to 36 (Table-1) [12]. The chicks were treated from day 1 to day 36 of the experiments. The chitosan used in this investigation was a 200-nm commercial shrimp shell chitosan (CV. ChiMultiguna, Indonesia).

**Table 1:** Broiler feed composition.

Ingredients (%, unless otherwise specified)	Starter (day 8–21)	Finisher (day 22–36)
Yellow maize	54.1	62.7
Palm oil	2.10	2.10
Soybean meal	40.0	31.7
DL-methionine	0.19	0.19
Bentonite	0.75	0.75
Limestone	0.75	0.75
Monocalcium phosphate	1.30	1.05
Premi×^1^	0.34	0.34
Chlorine chloride	0.07	0.07
Salt	0.40	0.40
Nutritional compositions		
ME (kcal/kg)^2^	2,900	3,004
Crude protein, %	22.0	19.0
Crude fiber, %	5.50	5.62
Ca, %	1.04	0.96
P (available), %	0.57	0.54
Met, %	0.52	0.48
Lys, %	1.18	0.98

^1^Each kg of feed contained 1,100 mg Zn, 1,000 mg Mn, 75 mg Cu, 850 mg Fe, 4 mg Se, 19 mg I, 6 mg Co, 1,225 mg K, 1,225 mg Mg, 1,250,000 IU vit A, 250,000 IU vit D_3_, 1,350 g pantothenic acid, 1,875 g vit E, 250 g vit K_3_, 250 g vit B_1_, 750 g vit B_2_, 500 g vit B_6_, 2,500 mg vit B_12_, 5,000 g niacin, 125 g folic acid and 2,500 mg biotin, ^2^ME (metabolizable energy) was calculated based on the formula of Bolton [12]: 40.81 {0.87 [crude protein + 2.25 crude fat + nitrogen-free extract] + 2.5}

The chicks were reared in open broiler houses lined with rice husks as litter. During the experiment, a continuous lighting program was implemented. The infectious bronchitis virus and Newcastle disease virus (NDV) were sprayed on chicks as soon as they hatch to impart immunity. Using drinking water, the chickens were vaccinated against NDV (Medivac ND La Sota, Indonesia) on day 18 after receiving the Gumboro (infectious bursal disease virus/IBDV) vaccination at age of 11 days (Medivac Gumboro B vaccine). Weekly body weight, feed consumption, and feed conversion ratio (FCR) measurements were made. A 3 mL syringe was used to collect blood sample from wing vein of one male chick (representing the average body weight for each replicate) per replicate (seven chicks per treatment group) on day 36. Blood was placed in a vacutainer containing an anticoagulant (ethylenediaminetetraacetic acid/ethylenediaminetetraacetic acid) for complete blood count analysis. In the absence of anticoagulants, the remaining blood was transferred to another vacutainer. The blood was allowed to clot at 25°C for approximately 2 h before centrifugation for 10 min at 500 × *g* to produce serum. The serum was stored in a freezer at 10°C until analysis. The intestines were removed from the chicken as soon as it was slaughtered. To measure the morphology of the small intestine (villus height and crypt depth [CD]), segments of the duodenum, jejunum, and ileum (approximately 2 cm) were placed in 10% buffered formalin (Leica Biosystems Richmond, Inc., Richmond, USA). The ileum and cecum digesta were placed in each sterile sample pot and subjected to additional laboratory analysis to determine the population of selected bacteria in the intestines.

### Laboratory analysis

Complete blood counts were measured using the Prima Fully-Auto Hematology Analyzer (PT. Prima Alkesindo Nusantara, Jakarta, Indonesia) according to the manufacturer’s protocols. Enzyme-based colorimetric techniques were used to determine serum lipid profiles (total triglycerides, total cholesterol, low-density lipoprotein, and high-density lipoprotein), as well as serum creatinine and uric acid levels. The levels of serum total protein, albumin, glucose, and aspartate aminotransferase were determined using spectrophotometric and photometric techniques. To determine the globulin concentration, the total protein value was subtracted from the serum albumin value. All biochemical analyses of serum samples (DiaSys Diagnostic System GmbH, Holzheim, Germany) were performed according to the manufacturer’s instructions.

Hematoxylin and eosin-stained 5 μm sections of the duodenum, jejunum, or ileum were used to histologically examine the small intestinal segments. The villous height and CD in each segment were measured using an optical microscope fitted with a digital camera (Leica Microsystems GmbH, Wetzlar, Germany). For each sample, the mean CD and villous height were determined using five measurements. The bacterial population in the ileal and cecal contents was determined using the total plate count method. Coliforms and lactose-negative *Enterobacteriaceae* were counted as red and colorless colonies on MacConkey agar (Merck KGaA, Darmstadt, Germany) after a 24-h aerobic incubation at 38°C. Lactic acid bacteria (LAB) were counted on de Man, Rogosa, and Sharpe (MRS; Merck KGaA) agar after a 48-h anaerobic incubation period at 38°C.

### Statistical analysis

Statistical Package for the Social Sciences version 16.0 (IBM Corp., NY, USA) was used to analyze the data collected during the study. Duncan’s multiple analysis was performed when there was a discernible effect (p= 0.05) from the treatments. Tendency was implemented when 0.05 ≤ p < 0.10.

## Results

Table-2 presents data on the final body weight, cumulative feed consumption, and FCR of broilers. There was a tendency (p = 0.08) for EACH bird to have a higher final body weight than the other broiler groups. Cumulative feed consumption was higher (p < 0.05) in EACH broilers than in EALE and CHIT broilers; however, it did not differ from that in CNTL broilers. FCR was lower (p < 0.05) in EALE, CHIT, and EACH broilers than in CNTL broilers.

**Table 2:** Final body weight, cumulative feed consumption and FCR of broilers.

Parameters measured	CNTL	EALE	CHIT	EACH	SEM	p-value
Final body weight, g/bird	1758	1799	1829	1870	16.2	0.08
Cumulative feed consumption, g/bird	2895^ab^	2828^b^	2854^b^	2954^a^	85.8	0.04
FCR	1.65^a^	1.57^b^	1.56^b^	1.58^b^	0.01	0.01

^a,b^With divergent superscripts, the means within the similar row differ considerably (p < 0.05), CNTL=Basal feed with no additives, EALE=Basal feed with 0.01% encapsulated *Acalypha australis* L. leaf extract, CHIT=Basal feed added with 0.01% chitosan, EACH=Basal feed with 0.01% encapsulated *Acalypha australis* L. leaf extract and 0.01% chitosan, FCR=Feed conversion ratio, SEM=Standard error of the means

The number of erythrocytes in the EALE group was lower (p < 0.05) than that in the CNTL and EACH groups (p < 0.05), but it was not significantly different from that in the CHIT groups. The hematocrit values were lower (p *=* 0.05) in the EALE and CHIT groups than in the other two treatment groups. There was a clear tendency (p = 0.09) for the number of heterophils to be higher in EACH group than in the CNTL group. The thrombocyte counts were lower (p < 0.05) in this group than in the other treatment groups. The complete blood count data of broilers are presented in Table-3.

**Table 3:** Complete blood counts of broilers.

Parameters measured	CNTL	EALE	CHIT	EACH	SEM	p-value
Erythrocytes (10^12^/L)	1.84^a^	1.67^b^	1.72^ab^	1.84^a^	0.03	0.02
Hemoglobin (g/dL)	7.29	7.13	6.91	7.36	0.09	0.35
Hematocrits (%)	31.0^a^	27.9^b^	28.4^b^	31.3^a^	0.46	0.01
Leukocytes (10^9^/L)	65.4	63.5	61.7	67.8	1.41	0.48
Heterophils (10^9^/L)	1.80	2.37	2.06	2.90	0.16	0.09
Lymphocytes (10^9^/L)	63.6	61.0	59.6	64.9	1.38	0.54
Thrombocytes (10^9^/L)	93.7^ab^	76.7^bc^	112^a^	58.3^c^	5.84	<0.01

^a,b,c^With divergent superscripts, the means within the similar row differ considerably (p < 0.05), CNTL=Basal feed with no additives, EALE=Basal feed with 0.01% encapsulated *Acalypha australis* L. leaf extract, CHIT=Basal feed with 0.01% chitosan, EACH=Basal feed with 0.01% encapsulated *Acalypha australis* L. leaf extract and 0.01% chitosan, SEM=Standard error of the means

The serum biochemical parameters of broiler chickens are listed in Table-4. Serum globulin levels were clearly higher (p < 0.05) in EACH than in CNTL and CHIT but did not differ from those in EALE. The albumin-to-globulin ratio was higher (p < 0.05) in CNTL broilers than in EALE and EACH broilers which was consistent with that observed for CHIT broilers.

**Table 4:** Serum biochemical indices of broilers.

Parameters measured	CNTL	EALE	CHIT	EACH	SEM	p-value
Total cholesterol (mg/dL)	112	122	101	130	5.99	0.38
Total triglyceride (mg/dL)	91.7	84.4	77.5	126	9.92	0.33
LDL (mg/dL)	28.4	33.6	25.2	36.1	3.16	0.63
HDL (mg/dL)	65.3	71.6	67.1	68.6	2.49	0.85
LDL/HDL	0.43	0.49	0.38	0.54	0.05	0.68
Total protein (g/dL)	2.96	3.31	2.80	3.45	0.12	0.22
Albumin (g/dL)	1.30	1.31	1.16	1.34	0.04	0.52
Globulin (g/dL)	1.61^b^	2.00^ab^	1.64^b^	2.25^a^	0.09	0.03
Albumin/globulin	0.78^a^	0.66^b^	0.73^ab^	0.65^b^	0.02	0.04
Uric acid (mg/dL)	3.27	2.23	2.79	4.06	0.35	0.31
Creatinine (mg/dL)	0.05	0.05	0.04	0.08	0.01	0.10
AST (g/dL)	266	248	236	296	11.8	0.30

^a,b^With divergent superscripts, the means within the similar row differ considerably (p < 0.05), CNTL=Basal feed with no additives, EALE=Basal feed added with 0.01% encapsulated *Acalypha australis* L. leaf extract, CHIT=Basal feed added with 0.01% chitosan, EACH=Basal feed with 0.01% encapsulated *Acalypha australis* L. leaf extract and 0.01% chitosan, LDL=Low-density lipoprotein, HDL=High-density lipoprotein, A/G ratio=Albumin-to globulin ratio, AST=Aspartate aminotransferase, SEM=Standard error of means

There was a strong tendency (p = 0.05) for coliform bacterial counts to be lower in the cecum of EACH broilers than in other broiler groups. Similarly, the ratio of LAB to coliform bacteria tended to be higher (p = 0.08) in the cecum of EACH broilers than in the other treatment groups (Table-5). The selected bacterial population did not differ (p > 0.05) in the broiler ileum among the dietary treatment groups.

**Table 5:** Selected bacterial population of the intestines of broilers.

Parameters measured	CNTL	EALE	CHIT	EACH	SEM	p-value
Ileum						
LAB (log cfu/g)	5.44	5.58	5.08	5.60	0.22	0.85
Coliform (log cfu/g)	2.84	2.52	3.30	2.50	0.22	0.57
LNE (log cfu/g)	2.08	2.00	2.47	2.21	0.09	0.27
LAB/coliform	2.26	2.31	1.66	2.44	0.15	0.24
Cecum						
LAB (log cfu/g)	8.50	8.63	8.35	8.10	0.12	0.43
Coliform (log cfu/g)	5.42	4.98	5.66	4.39	0.18	0.05
LNE (log cfu/g)	3.79	3.51	4.00	3.19	0.17	0.37
LAB/coliform	1.59	1.78	1.48	1.93	0.07	0.08

CNTL=Basal feed with no additives, EALE=Basal feed added with 0.01% encapsulated *Acalypha australis* L. leaf extract, CHIT=Basal feed added with 0.01% chitosan, EACH=Basal feed with 0.01% encapsulated *Acalypha australis* L. leaf extract and 0.01% chitosan, LAB=Lactic acid bacteria, LNE=Lactose-negative *Enterobacteriaceae*, cfu=colony forming units, SEM=Standard error of means

Small intestinal morphology data are presented in Table-6. No substantial influence (p > 0.05) of the dietary treatments on the villi height (VH), CD, and the ratio of villus height and CD across the small intestinal segments (duodenum, jejunum, and ileum) of broiler chickens was observed.

**Table 6:** Morphology of the small intestinal segments of broilers.

Parameters measured	CNTL	EALE	CHIT	EACH	SEM	p-value
Duodenum						
VH (µm)	1198	1255	1144	1164	41.9	0.82
CD (µm)	261	244	257	274	11.6	0.85
VH/CD	4.75	5.35	4.55	4.61	0.24	0.66
Jejunum						
VH (µm)	831	1039	844	897	50.2	0.46
CD (µm)	194	217	208	212	12.8	0.93
VH/CD	4.47	4.89	4.41	4.70	0.30	0.95
Ileum						
VH (µm)	573	498	525	611	48.4	0.87
CD (µm)	157	145	163	174	8.62	0.69
VH/CD	5.11	4.48	4.06	3.71	0.27	0.30

CNTL=Basal feed with no additives, EALE=Basal feed added with 0.01% encapsulated *Acalypha australis* L. leaf extract, CHIT=Basal feed added with 0.01% chitosan, EACH=Basal feed with 0.01% encapsulated *Acalypha australis* L. leaf extract and 0.01% chitosan, VH=Villi height, CD=Crypt depth, SEM=Standard error of means

## Discussion

The data in this study showed that the use of EALE, chitosan, or a combination of both improved the FCR of broiler chickens during rearing when compared with control. Another finding was that chickens treated with a combination of EALE and chitosan had a higher final body weight than control chickens. No increase in body weight was observed in chickens treated with EALE or chitosan alone. These results indicate the synergy between the EALE and chitosan in promoting broiler chicken growth rate. Chitosan increased the ability of chickens to absorb and utilize the active compounds in *A. australis* leaf extract [11], thus improving the physiological conditions and growth performance of broiler chickens. With regard to cumulative feed consumption, the administration of EALE and chitosan resulted in higher feed consumption than the administration of EALE or chitosan individually. To date, the reason for the high feed consumption in this group of broiler chickens has not been known. It has been shown that feed intake is greatly influenced by the immunological condition of broiler chickens. Klasing *et al*. [13] reported that inflammation decreases feed intake, disrupts the morphology of the intestine, obstructs the absorption of nutrients, and modifies skeletal muscle anabolic processes so that nutrients can be used for immune function. Our inference was supported by the data of thrombocytes in our present study, at which the level of thrombocytes was lower in broilers receiving EALE and chitosan than in chickens receiving EALE or chitosan individually. It should be noted that thrombocytes are an indicator of the inflammatory status in poultry, where thrombocytes increase in birds experiencing inflammation [14]. With respect to the control group, whose feed intake was the same as that of the other three groups, it was highly probable that the elevated thrombocyte counts in this group influenced alterations in anabolic processes, thereby increasing the amount of nutrient used for immune function. This condition was demonstrated by the fact that feed utilization was less efficient (higher FCR value) in the control group than in the chickens in the combination of EALE with chitosan.

Broiler chickens receiving EALE had lower levels of erythrocytes in the blood than chickens in other treatment groups. Adegoke *et al*. [15] reported that the phenol content in herbal products can reduce the absorption of iron from feed. Considering that iron is responsible for the production of hemoglobin, reduced iron can reduce the value of hemoglobin and erythrocytes in the circulatory system of broilers. However, this inference should be taken with caution because hemoglobin values did not significantly differ among treatment groups in the current study. In general, hematocrit calculates the volume of erythrocytes in relation to the total volume of the blood. Therefore, hematocrit values are greatly influenced by the number and size of erythrocytes. Consistent with the above-mentioned theory, this study showed lower levels of hematocrit in broilers receiving EALE or chitosan, which agreed with the lower levels of erythrocytes in both groups of chickens. The number of heterophiles was higher in the blood of broiler chickens treated with EALE and chitosan than in control chickens. An increase in the number of heterophils is often associated with an increase in bacterial infections in broiler chickens because heterophils act as the first line of innate immunity in chickens [16]. However, this assumption cannot be accepted in the present study because the number of pathogenic bacteria (coliforms) in the cecum of broiler chickens that received a combination of EALE and chitosan was actually lower than that in other chickens. In addition, there were no differences in the pathogenic bacteria counts in the ileum of broilers between the treatment groups. Therefore, the increase in the number of heterophils in chickens that received a combination of EALE and chitosan was not related to the bacterial load in these chickens. It is very possible that the increase in the number of heterophils is a positive impact of the synergy between EALE and chitosan, thereby positively improving the immune system in broiler chickens. In this study, increasing the number of heterophils had an impact on reducing the bacterial load in the digestive tract of broiler chickens, which can be seen in the reduction in the number of coliforms in the cecum of broiler chickens. As discussed above, an increase in the number of thrombocytes is an indicator of inflammation in broiler chickens, one of which is due to bacterial infection [14]. In the present study, chickens that received a combination of EALE and chitosan had a lower number of thrombocytes than chickens that received a combination of EALE and chitosan. This result indicated an improvement in immune function in chickens treated with this combination therapy.

Increased serum globulin levels in broiler chickens are typically attributed to improved immune function [17]. In this study, serum globulin levels were highest in broilers that received a combination of EALE and chitosan. These conditions may lead to enhanced immune defense function and lower pathogenic load in broilers. The lower coliform counts in the cecal digesta and the lower thrombocyte levels (one of the inflammatory mediators) supported the latter conclusion. It is not known if the improved immune competence of birds receiving a blend of EALE and chitosan has improved. However, complementary or synergistic effects between EALE and chitosan appear to be responsible for the enhanced immune competence of broilers. In this study, both herb [5, 7] and chitosan [10] exhibited immune-enhancing effects on broiler chickens. A lower albumin-to-globulin ratio indicates improved protein metabolism and immune functions in broilers [18]. The results showed that broilers receiving EALE or a combination of EALE and chitosan had a lower albumin-to-globulin ratio than the control. These results indicated that the immune properties of the broiler groups improved.

Although there was no discernible influence of the dietary treatments on the selected bacterial population in the ileum, the number of coliforms in the cecum decreased with dietary supplementation of *A. australis* leaf extract and chitosan blends. The antimicrobial properties of *A. australis* leaf extract [3] and chitosan [9, 10] synergistically work against pathogenic bacteria in the gastrointestinal tract of broiler chickens. It appears that the latter has a greater antibacterial effect on the gastrointestinal tract of broilers than either EALE or chitosan. In this study, blends of chitosan and EALE showed a tendency to increase the LAB to coliform ratio in the cecum of broiler chickens. The increased ratio of LAB to coliforms in the digestive tract of broilers suggests that LAB predominate over coliforms. A higher ratio of LAB to coliforms confers greater resistance to intestinal disease in broiler chickens [19]. Increases in the ratio of LAB to coliforms may also improve immune function in broiler chickens. The elevated LAB to intestinal coliform ratio resulting from the blends of EALE and chitosan was consistent with the superior immunological competence of broiler chickens, as evidenced by the elevated globulin levels in these chickens.

The results of the current study did not show any effect of treatments on the small intestinal morphology of broilers. This finding differs from Sugiharto’s [7], suggesting that the use of herbal ingredients in feed can improve the intestinal morphology of broilers. The use of chitosan as a feed additive has also been reported to increase VH and villus height-to-CD ratio, leading to an improvement in broiler growth performance [10]. It is unknown why the treatment used in this study did not significantly alter the intestinal morphology of broilers. However, the lack of any effect of dietary treatment on microbes in the small intestine may account for the lack of effect of the treatment on intestinal morphology. A variety of factors influence the morphology of the small intestine, including the population of microbes in the small intestine of broilers [1]. This study found that dietary treatment significantly increased feed utilization efficiency, but intestinal morphology remained unchanged. The efficiency with which feed was used and the growth of broiler chickens are likely to benefit from the improvement of protein metabolism resulting from dietary supplementation, as evidenced by a decrease in albumin-to-globulin ratio [18].

## Conclusion

A combination of EALE and chitosan as feed additives enhanced the final body weight and feed efficiency (FCR) of broilers. These additives also increased the levels of heterophils, serum globulin, the ratio of LAB to coliforms, and reduced thrombocytes, albumin-to-globulin ratio, and cecal coliform bacteria. Hence, EALE and chitosan blend improved the growth performance, immune status, and intestinal health of broiler chickens.

## Authors’ contributions

SS: Designed the study, analyzed the data, drafted the manuscript, and obtained funding for this study. YZ, IA, EW, HIW, TY, and TAS: Performed the experiments and laboratory analyses. All authors have read, reviewed, and approved the final manuscript.
